# Sex and Brain: The Role of Sex Chromosomes and Hormones in Brain Development and Parkinson’s Disease

**DOI:** 10.3390/cells12111486

**Published:** 2023-05-27

**Authors:** Francesca Terrin, Annachiara Tesoriere, Nicoletta Plotegher, Luisa Dalla Valle

**Affiliations:** Department of Biology, University of Padova, 35131 Padova, Italy; francesca.terrin@phd.unipd.it (F.T.); annachiara.tesoriere@phd.unipd.it (A.T.)

**Keywords:** sex hormones, sex chromosomes, neurodegeneration, Parkinson’s disease, brain development, sexual differentiation

## Abstract

Sex hormones and genes on the sex chromosomes are not only key factors in the regulation of sexual differentiation and reproduction but they are also deeply involved in brain homeostasis. Their action is crucial for the development of the brain, which presents different characteristics depending on the sex of individuals. The role of these players in the brain is fundamental in the maintenance of brain function during adulthood as well, thus being important also with respect to age-related neurodegenerative diseases. In this review, we explore the role of biological sex in the development of the brain and analyze its impact on the predisposition toward and the progression of neurodegenerative diseases. In particular, we focus on Parkinson’s disease, a neurodegenerative disorder that has a higher incidence in the male population. We report how sex hormones and genes encoded by the sex chromosomes could protect from the disease or alternatively predispose toward its development. We finally underline the importance of considering sex when studying brain physiology and pathology in cellular and animal models in order to better understand disease etiology and develop novel tailored therapeutic strategies.

## 1. Sex Hormones and Sex Chromosomes as Players in the Sexual Differentiation of the Mammalian Brain

Sex differences in the human brain, both at the morphological and physiological levels, have always been an interesting and crucial research theme for the scientific community, not only for their correlation with cognition, learning capacity, and reproductive behavior but also for the implications that these differences could have in relation to pharmacological effects of drugs and disease susceptibility, including their relevance in the differential occurrence of neurodegenerative pathologies.

In recent years, more and more research has been conducted to shed light on the mechanisms that regulate brain sexual differentiation, a process that, in mammals, occurs starting from the early development of the embryo and extends through critical prenatal and perinatal periods. Sexual differentiation affects brain morphology and physiology, and the resulting sex differences are then maintained and reinforced through puberty and adult life.

The main forces that drive brain sexual differentiation comprehend the hormonal “milieu”, which is built up by early gonadal steroid hormone secretion [1], genetic contribution of genes encoded by the sex chromosomes [2,3], and long-term and short-term epigenetic regulation of gene expression modulated by gonadal hormones and their metabolites [4,5].

### 1.1. Steroid Hormones in the Central Nervous System

The central nervous system (CNS) has long been recognized as one of the main targets of the action of steroid hormones. In the brain, steroid hormones exert pleiotropic effects since they regulate many functions, including neurogenesis and neuroprotection [6,7].

Steroid hormones are produced by steroidogenic glands (gonads, adrenal glands, and placenta) and transported, bound to their partner transport proteins, through the circulatory system to the brain. Steroids can easily cross the blood–brain barrier to reach their target sites in nervous tissues. In addition, different steroid hormones can be synthesized in the central and peripheral nervous systems starting from cholesterol or steroid precursors generated in peripheral sources. These locally synthesized hormones can act through paracrine, autocrine, and intracrine communication mechanisms. The seminal discovery of the brain’s steroidogenic potential and the term “neurosteroids” to describe hormones produced therein were first reported in 1981 by the Baulieu and Robel group. They demonstrated how some steroids (such as pregnenolone, dehydroepiandrosterone (DHEA), and their sulfate esters) are present at higher concentrations in the brain than in the plasma of adult male rats, and persist after surgical excision of the classical steroidogenic glands (reviewed in [8]). The nervous system is then provided with the steroidogenic enzymes required to synthesize all classes of steroids and, thus, constitutes a target for hormonal steroids as well as neurosteroids produced by neurons and glial cells.

While the term “neuroactive steroids” comprises all steroids active in the brain, including those coming from the circulation, neurosteroids are limited to those directly synthesized or activated in the brain from peripheral sterol precursors (reviewed in [9]). Neurosteroids can work as endogenous regulators of neuronal functions and excitability, mainly via rapid non-genomic mechanisms [10]. Based on their structural characteristics, neurosteroids can be classified as pregnane, androstane, and sulfated neurosteroids [11]. Generally, neurosteroids modulate brain excitability primarily by interacting with neuronal membrane receptors and ion channels, principally GABA-A receptors [12].

**Figure 1:**
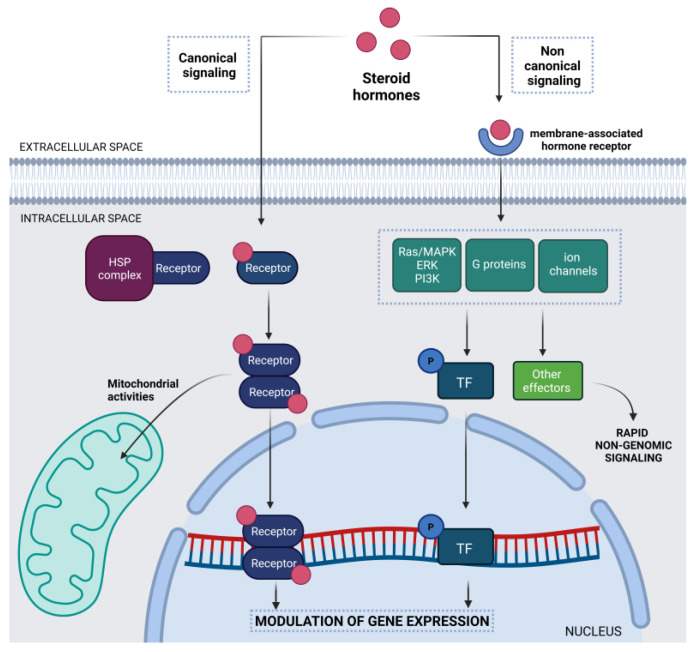
Steroid hormones can act through both canonical and non-canonical signaling. In canonical signaling, they bind and activate their cognate hormone receptors that dimerize, enter the nucleus, and bind to specific sites on DNA, thereby modulating gene expression. In the non-canonical signaling pathways, hormones bind membrane-associated receptors, thus activating different kinase cascades that rapidly induce a non-genomic response. Alternatively, they can indirectly modulate gene expression through the phosphorylation (P) of transcription factors that bind DNA. HSP complex: heat shock protein complex, ERK: extracellular signal-regulated kinase, PI3K: phosphoinositide 3-kinase, TF: transcription factor. Figure created with BioRender.com.

The effects of neuroactive steroids, neurosteroids, and sex hormones working in the brain are mediated not only by classical nuclear signaling (genomic action through intracellular steroid receptors) but also by non-classical rapid signaling via binding to membrane-associated receptors or transmembrane receptors (Figure 1).

The canonical/classical mechanism of action involves intracellular binding of steroids to specific receptor proteins located in the cytoplasm or the nucleus, which belong to the nuclear receptor superfamily and act as transcription factors. Circulating steroids diffuse through the plasma membrane and bind to their cognate receptors. This process induces the release of the receptors from heat shock proteins and their conformational change and activation. Once activated, the complex of steroid hormone/receptor translocates into the nucleus, where it can bind hormone-responsive elements (HREs) in the promoter regions of target genes, thereby regulating gene expression [13]. The nuclear activity of steroid receptors can induce or repress the transcription of target genes involved in neural differentiation or neuronal functions [14]. These hormonal effects can also influence animal and human behaviors during development and adulthood, particularly sexual behavior [15] (Table 1).

Steroid hormonal effects are also finely modulated through the action of co-activators in the nucleus [16] or by the activation of rapid signaling pathways [17]. In this case, steroid hormones act through membrane-associated receptors in different ways based on the type of steroids (Table 2).

Other non-canonical mechanisms of action of steroid hormones could be identified in the mitochondria of neuronal cells of the nervous system. The presence of putative HREs in the mitochondrial genome suggests a possible role in regulating the transcription of mitochondrial genes [18]. In general, both the direct and non-direct effects of steroid hormones on mitochondrial metabolism have been found to be involved in their neuroprotective effects [19].

**Table 1:** Canonical signaling of steroid hormones in the CNS.

Steroid Hormone	Activated Receptor	Functions	Targets	References
Estrogen	ERα, ERβ	Neuroprotection	Antiapoptotic genes	[20,21,22,23]
			Proliferative genes	
Progesterone	PR-A, PR-B	Neuroprotection	Promyelinization genes	[24,25]
			Anti-inflammatory genes	
Testosterone	AR	Neuroprotection	Remyelination process by oligodendrocytes	[26,27,28]
			Astrocyte recruitment	
		Sex phenotype	Development and maintenance of male phenotype in CNS	[29,30,31]

ERα: estrogen receptor alpha, ERβ: estrogen receptor beta, PR-A: progesterone receptor A, PR-B: progesterone receptor B, AR: androgen receptor.

**Table 2:** Non-canonical signaling of steroid hormones in the CNS.

Steroid Hormone	Activated Receptor	Functions	References
Estrogen	ERα, ERβ, mERs, GPER1	Oligodendrocyte differentiation, survival, and function	[32]
		Spine plasticity	[33,34,35,36]
		Behavior	
Progesterone	PR-A, PR-B, mPRs, PGRMC1	Female reproductive behavior	[37,38,39,40]
		Cerebellum cortical formation	
		Neuroprotection	
Testosterone	AR, GPCRs	Sexual differentiation	[41,42,43,44,45,46]
		Reproductive and aggressive behaviors	
		Neuroendocrine response	

mERs: membrane-associated estrogen receptors, GPER1: G protein-coupled estrogen receptor 1, mPRs: membrane-associated progesterone receptors, PGRMC1: progesterone receptor component 1, GPCRs: G protein-coupled estrogen receptor.

### 1.2. Sex Hormones’ Modulation of Brain Sexual Differentiation

In mammals, gonadal sex determination is directed by the presence or absence of the Y chromosome containing the *Sex-determining Region Y* gene (SRY) that encodes the “testis determining factor”. This is a transcription factor working as a molecular switch to activate Sertoli cell differentiation and the development of the testis. In the absence of this protein, the bipotential and undifferentiated gonad gives rise to an ovary [47,48].

The testis, then, starts to produce testosterone, which regulates the development of male sex organs, reproductive tract, and secondary sexual characteristics. In contrast to the steroidogenic activity already present in fetal Leydig cells of the testis, no hormones are produced by the developing ovaries, and the female sex organs progress without signals. The molecular mechanisms that control mammalian gonadal development are beyond the focus of this review but are fully described in many previous review papers [49,50,51].

**Figure 2:**
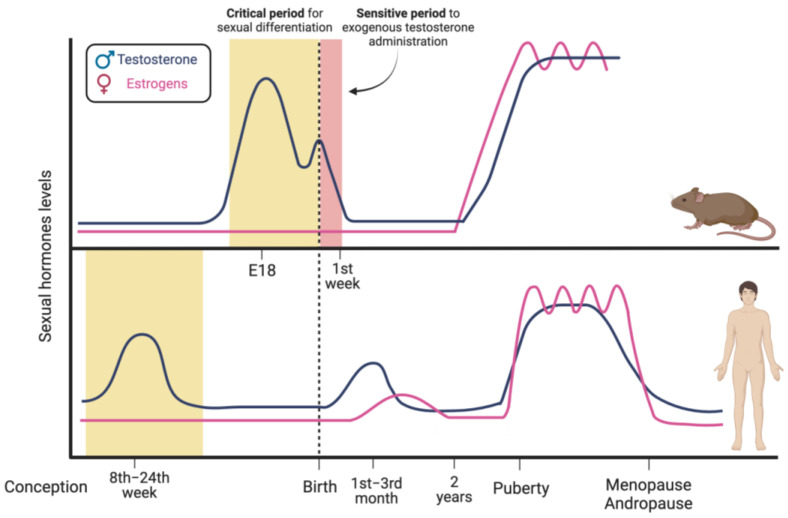
The sexual differentiation of the brain is hormonally induced and occurs during the “critical period”, a limited sensitive temporal window. The **upper panel** shows the two peaks of testosterone in male rodents that masculinize the brain (organizational activity). In the absence of testosterone and, consequently, of estrogen, feminization of the brain takes place. At puberty, the gonads start to produce corresponding male and female sex hormones to activate the brain structures organized during development and mediate sexually dimorphic behaviors. In humans (**lower panel**), the level of fetal steroid testosterone peaks between weeks 11 and 15, and, thus, the sensitive period occurs prenatally. The panel describes the changes in the levels of circulating androgens and estrogens during lifetime in humans. Created with BioRender.com.

Sex-related processes underlying brain sexual differentiation have been mainly studied via exploiting rodents as animal models. In male rodents, testosterone is produced by fetal Leydig cells and first peaks during late gestation, which is around embryonic days 17–19, while a second lower peak occurs within a few hours after birth (Figure 2, upper panel) [52,53].

In accordance with the “Organization and Activation Hypothesis” [54], sex differences in brain physiology and functions are initially defined by exposure of the brain to testosterone secreted during the so-called “critical period”, a precise temporal window of development in which the brain is particularly sensitive to estrogens produced by the neural aromatization of testosterone [1,54,55,56]. The aromatase enzyme, which is responsible for this conversion, is expressed by neurons of specific brain regions, such as the hypothalamus, the preoptic area, and the limbic system, which control not only the reproductive axis, but also the processing of sensory information, affective behavior, and the modulation of learning and memory [57,58,59].

The “critical period” represents a constant in the process of brain differentiation in mammalian species examined so far, even if its temporal positioning during development is variable [56]. The extraglandular synthesis of “neurosteroids” has also been reported in some regions of the developing brain, thus resulting in variable steroid content depending on the brain area and sex [60].

Therefore, the normal development of male rodent brain undergoes the process of masculinization, in which increased perinatal levels of estradiol orchestrate the formation of neuronal circuits, the synthesis of neurotransmitters, and the number, architecture, and connectivity of nerve cells, ultimately defining the morphological and size differences that exist among specific regions of the brain in males and females [4,56]. At the same time, male rodent brain undergoes the steroid-induced process of defeminization, which consists in the loss of the ability to display female-typical behaviors [61].

Female rodents, on the contrary, are protected from brain masculinization by the plasma glycoprotein α-fetoprotein, which binds circulating estradiol of maternal or placental origin, thus preventing it from entering the brain [62].

In male rodents, the critical period extends to the first two postnatal days, while females are sensitive during the first week after birth. From an experimental point of view, this larger postnatal window allows the easy execution of experiments to validate the hypothesis of the fundamental role of hormones in brain development. For example, if testosterone is administered to newborn female rats, thus mimicking the hormonal stimulation by fetal testis that occurs in males, the brain is masculinized. Consequently, these animals exhibit male-typical sexual behaviors, such as mounting behavior, despite the presence of the ovary and XX genotype. On the other hand, stimulation with androgen outside this sensitive time interval does not affect sexual brain differentiation [63].

Females are not exposed to endogenous steroids since the fetal ovaries are functionally quiescent. This lack of steroidogenic activity suggests the possibility that the brain acquires female morpho-functional characteristics in the absence of specific hormones, following a “default program”. However, previous experiments performed with aromatase knockout female mice and post-natal treatments with estradiol suggest that this hormone may contribute to the development of female sexual behaviors [64,65] and that brain plasticity and sensitivity to the organizational effects of steroid hormones persist longer than previously thought.

Hormones produced by the fetal testis act on the developing brain during the so-called “organizational” phase, in which modifications of the cerebral transcriptional profile determine the development of neural circuits responsible for sex-specific behaviors in subsequent periods of life [66].

After puberty, hormones produced by the gonads will instead carry out what are defined as “activation effects” on the brain circuits that are previously set in a sex-specific sense [67].

In humans, testosterone starts to be produced by the testis at the 8th week after conception, it peaks between weeks 11 and 15 and then declines to reach low levels at the 24th week [68,69]. Consequently, the period that is sensitive to the hormonal environment and sexual differentiation of the brain precedes birth [70].

Male newborns show a second, lower, and time-restricted peak around three months of life, while females show an increase in estradiol that then declines very slowly during the first two years of life. This hormonal situation is known as “mini-puberty” [71] and could contribute to the sexual organization of the brain in humans. Testosterone levels rise again at puberty and are maintained during the reproductive adult age (activational period). They finally tend to decrease with aging (Figure 2, lower panel) [72].

Unlike what has been shown in rodents, the masculinizing agent in humans is represented by androgens, as also demonstrated by males in which the aromatase enzyme or the ERα are not functional. These subjects identify themselves as males, suggesting that the differentiation likely depends on androgens [73,74,75].

To date, knowledge of the mechanisms that lead to brain differentiation in humans comes from the study of the so-called differences (or disorders) of sex development (DSD), which include congenital conditions linked with atypical progress of internal and external genital structures. As an example, in the Androgen Insensitivity Syndrome (AIS), the testis present in XY individuals produces testosterone but cannot transduce its signal due to the lack of a functional AR. These individuals not only have a female phenotype but also recognize themselves as females and exhibit female behaviors and brain morphology, in accordance with the fundamental role of androgens on the sexual differentiation of the brain [76,77].

### 1.3. Sex Hormone-Dependent Brain Sexual Dimorphism

The role of steroid hormones in the process of brain sexual differentiation and in the determination of structural dimorphism of some specific regions and nuclei has been known for a long time. As a main example, the medial preoptic area (MPOA) of the hypothalamus that controls male sexual behaviors and gonadotropin release is 3- to 5-fold larger in rodent males than females [66], and its neurons contain twice the number of dendritic spines than the neurons in the female brain [78]. This dimorphism seems to be related to testosterone regulation of the apoptotic process occurring in this area [79].

Moreover, the posterior bed nucleus of the stria terminalis (BNSTp) is a sexually dimorphic area that appears larger in males than females because of its male-biased gene expression and neuronal development driven by estrogens, which promote neuron survival in this area [80]. On the other hand, the anteroventral periventricular nucleus (AVPV), which is involved in the control of ovulation [66] and gonadotropin secretion [81], is larger in females than in males [63,66,82]. These structural differences relate to the rates of neurogenesis, cell migration and differentiation, axon guidance, synaptogenesis, and cell death [83], which can be regulated by estrogens binding to both nuclear and membrane receptors. Estrogens induce a higher rate of cell survival in male MPOA and an increase in apoptosis in the same region of the female developing brain. On the contrary, estradiol promotes cell death in male AVPV, leading to the aforementioned sex-dimorphic difference in this region. These opposite effects of estrogen indicate that cellular responses to the same gonadal hormone are sex-specific and differ among specific areas of the CNS [66].

Estrogen receptors exist in two isoforms, ERα and ERβ, which are encoded by the *ESR1* and *ESR2* genes and are widely expressed in the mammalian brain. They show a different pattern of distribution [84] and time- and sex-specific expression and functions in the brain [84,85,86]. While ERα is mainly associated with the masculinization effect of estrogens on neuronal circuits and behavior, ERβ seems to be involved in the defeminization process of the male brain [61,66]. Consequently, a loss of ERα in males results in reduced sexual behavior, whereas knockout of *ERβ* alone has no effect on sexual behavior. However, when both ERs are dysfunctional, male sexual behavior is completely lost [87,88].

ERs have been reported to play a fundamental role in orchestrating cell movements in the developing brain. Estradiol can rapidly affect nerve cells’ migration via non-genomic transduction of its signal. Indeed, both ERα and ERβ can be expressed as membrane-bound proteins that, upon interaction with their ligand, can activate signal cascades involving classical second messengers, such as protein kinases and phosphatases, the release of cyclic amines, and calcium signaling [89]. Estrogens target GABAergic neurons, which play a critical role in hypothalamic development [83,90], interfere with BDNF (brain-derived neurotrophic factor) release in specific cell types [81], and are involved in a complex interplay with CREB (cyclic AMP response element-binding protein) that, in turn, can have an impact on neuron migration during brain development [83]. Moreover, estradiol can activate PI3 kinase and promote presynaptic glutamate release, thus affecting postsynaptic AMPA and NMDA receptors [91,92]. Interestingly, estrogen-mediated presynaptic glutamate and GABA release can permanently alter the synaptic profile of postsynaptic neurons and the morphology of neighboring astrocytes, respectively, even if these cells appear devoid of ERs. Thus, sex steroids interfere with brain cell organization and differentiation either locally or by exploiting cell–cell interactions in a domino fashion [66].

Of note, the alteration in the levels of BDNF and GABA, which are involved in neuronal development and signaling, and the following impairment of synaptic transmission have been recognized as a pathogenic mechanism of neurodegeneration [93].

Although estrogens appear to be a driving force shaping male rodent brains, androgens also contribute to sex-dependent dimorphism. Testosterone reaching the brain during the late gestation and the early postnatal period can either act directly or after being metabolized into its biologically more active form, dihydrotestosterone (DHT), by the isoform 2 of the enzyme 5α-reductase. The expression of this enzyme increases upon the surges in testosterone in the critical perinatal period, and, thus, it has higher levels in male brains than in females. Its localization and the timing of its expression suggest a role for DHT in brain sexual differentiation [94]. Nevertheless, it is expressed not only in late fetal and early postnatal brains but also in regions of adult rodent and human brains that are mainly involved in the regulation of behaviors [95].

Progesterone is another steroid hormone that is involved in brain development. It can be either synthesized in adrenal glands in males and females and in ovaries and placenta in females, but it can also be directly synthesized in the brain by neurons and glial cells [96], thus being considered a “neurosteroid” [97]. It interacts with intracellular PR, which acts as a nuclear transcription factor, with the putative membrane-associated PGRMC1, and with mPR. These interactions lead to the activation of JAK/STAT, Src pathways, protein kinase G, MAPK, and G protein cascades or to the modulation of GABAA receptors through progesterone bioconversion into allopregnanolone [97]. Progesterone levels in the brain show subtle sex-related differences, but the levels can vary substantially according to the cycle phase [98]. Several studies have indicated that the prenatal testosterone surge in males and its further conversion to estradiol indirectly induce the upregulation of PR expression in rodent sex-dimorphic brain regions, thus contributing to the masculinization of neuronal functions and sexual behaviors [99].

Although sex hormones are the main inducers of sexual dimorphism during the development of the brain, there is also evidence of the role played by other steroid hormones, in particular glucocorticoids, in the shaping of brain functions. These are mainly related to the correct development of the hypothalamic–pituitary–adrenal (HPA) axis that regulates, among others, behaviors and stress response [100].

### 1.4. Sex Chromosomes’ Impact on Brain Sexual Differentiation

The arousal of sex differences in the brain has been historically described as a hormonal-dependent brain organization that relates to the gonadal secretion of sex steroid hormones [83]. However, some sex differences cannot be explained by the activity of gonadal steroids. Other players in this process are the sex chromosomes, which are differentially inherited by male and female cells, resulting in differential gene expression and cell-autonomous effects [2,3].

The critical role played by the *Sry* gene in male sex determination has already been briefly described. However, besides its expression and function in the gonads, this gene also exerts many different functions in the brain. The work by Reinius and Jazin (2009), indeed, demonstrated the expression of Y-chromosome genes in many regions of the prenatal brain, suggesting their involvement in the sexual dimorphism of this organ [101].

For example, the *Sry* gene is expressed in the hypothalamus and midbrain of adult mice [102], as well as in the hypothalamus and frontal and temporal cortex of adult men, as demonstrated by an analysis of samples derived from autopsies [103], thus possibly exerting gonad-independent actions. Interestingly, *Sry* is also expressed in tyrosine hydroxylase (TH)-positive neurons within the *substantia nigra* (SN), where it can act as a positive regulator of dopamine biosynthesis and functions [66,104,105,106].

Differences linked to the sex chromosomes can also be associated with the differential imprinting of the genes deriving from maternal or paternal origin (reviewed in [107]), as well as by the dosage of the X-linked genes.

The female genome, indeed, contains two copies of the X chromosome, one of which will be inactivated into heterochromatin to re-balance the different genetic loads, a process called X-chromosome inactivation (XCI). Due to the presence of the two copies of this chromosome and the random inactivation process in each cell, females result in a mosaic of paternal and maternal active X chromosomes. This occurrence protects females from disorders due to mutation in the X-linked genes [108]. A fraction of the X-linked genes (15% in humans [109]) escapes inactivation and is expressed, albeit to a lesser extent, not only by the active X chromosome but also by the silent X chromosome. This could lead to higher expression levels of these genes in XX individuals compared to XY individuals, likely resulting in sex differences in female cells [108].

The influence of the sex chromosomes and their contribution to brain sexual differentiation have been experimentally verified by using four core genotypes (the FCG model), which permit the comparison between mice with similar gonads but different sex chromosomes [110,111]. The significant number of studies performed with these models allowed researchers to identify XX versus XY differences in behaviors, in gene expression, and in the predisposition to diseases (reviewed in [54]), including neurodegenerative diseases.

In humans, the effects of the sex chromosomes on brain anatomy have been studied on sex chromosome aneuploidies, such as the Turner syndrome (45X, female lacking one X chromosome) or the Klinefelter syndrome (47XXY, male with an additional X chromosome). Both syndromes present variations in the copy number of the sex chromosomes, allowing the analysis of possible gene dosage effects [112,113].

### 1.5. Epigenetic Regulation of Brain Sexual Differentiation

As previously described, steroid hormones are responsible for long-lasting organizational effects that sculpt the structure and functions of the developing brain and that, in many cases, are maintained throughout adulthood. These long-lasting effects can be mediated by the possible impact of steroid hormones on epigenetic mechanisms that lead to sustained changes in gene expression [4]. Epigenetic mechanisms, including posttranscriptional regulation by noncoding RNAs, histone modifications, and DNA methylation, can affect gene expression without altering the genetic code. Gonadal hormones, and estrogen in particular, may indeed regulate DNA methyltransferase (DNMT) expression and activity in the brain, thus exerting an important function in tuning chromatin accessibility [4].

As recently demonstrated by Gegenhuber and collaborators, ERα activation determined by the perinatal hormonal surge controls a sustained male-biased gene expression program in the developing brain, at least in the sexually dimorphic BNSTp region. Interestingly, chromatin is maintained in an active state by estradiol in both sexes, while testosterone can promote chromatin activation or repression in males through the action of ARs on AREs (Androgen Responsive Elements). However, only a small percentage of neonatal estradiol-regulated sex-biased gene expression is maintained during adulthood (about 10% of neonatal estradiol-regulated regions). Most of them undergo reprogramming and further regulation after puberty-induced hormonal changes, demonstrating how the genome maintains its plasticity and responsiveness to sequential variations in the hormonal milieu [80].

## 2. Sex-Related Differences in Neurodegeneration

In the previous section, we have largely discussed how sex hormones and sexual genes (on X and Y chromosomes) can regulate brain development, thereby producing a clear dimorphism. Recent research on the role of sex in brain development and physiology has generated an increasing interest in the possibility of having a sex-related predisposition or vulnerability to certain brain diseases, in particular neurodegenerative diseases. This hypothesis has largely been investigated, and it is actually strongly supported by the literature. In brief, biological sex seems to be connected to the incidence of neurodegenerative diseases.

Starting from these considerations, in this second section, we analyze sex-related differences in neuroinflammation and in neurodegeneration, focusing our attention on Parkinson’s disease (PD).

### 2.1. Focus on Parkinson’s Disease

PD is the second most common neurodegenerative disease (following Alzheimer’s disease), affecting about 3% of the population by the age of 65 and more than 5% of people over 85, and it is caused by the preferential loss of dopaminergic (DA) neurons in the *substantia nigra pars compacta* (*SNpc*) [114,115]. Degeneration of DA neurons leads to the onset of classic motor symptoms, including tremors at rest, bradykinesia, rigidity, and gait disturbances. Other non-motor symptoms may occur either in the early stages of the pathology, such as gastrointestinal disorders and sleep disturbances, or in the later stages when dementia and psychosis may appear [114].

The typical hallmark of this disease is the accumulation of intracellular proteinaceous inclusions in surviving neurons, which are named Lewy bodies and Lewy neurites, and are mainly constituted by the presynaptic protein α-synuclein (α-syn) [116]. The accumulation of α-syn aggregates can hamper autophagic flux and impact on various intracellular processes critical for neuronal functions, such as synaptic vesicle recycling and docking, finally leading to neuron death [117].

Another characteristic of this disease is sustained microglial activation that determines chronic neuroinflammation, thus contributing to neuronal loss [118].

Astrocytes have also been found to be activated in PD brains [119] and, together with microglia, are known to be involved in α-syn aggregate removal [120]. Despite neurons being the most affected cells in PD, alterations in glial cells are known to contribute extensively to PD etiology. Microglial function presents various sex-related features, which may be relevant for the sex-differences observed in PD that will be discussed below. PD is characterized as being sporadic (of unknown etiology) in about 90% of the cases, while it is associated with gene mutations in about 10% of patients. Among its non-genetic forms, PD has been shown to be associated with exposure to pesticides, such as paraquat and rotenone, or to other environmental pollutants [121,122], which are known to impact mitochondrial function and to induce oxidative stress, and that may increase the risk of developing PD.

The causative genes associated with PD or parkinsonism encode proteins involved in several pathways that are keys for the maintenance of brain homeostasis and functions. These genes are involved in the functioning of mitochondria, of the ubiquitin–proteasome and autophagic–lysosomal systems, in the regulation of oxidative stress and inflammation, as well as in the control of the homeostasis of synapses and synaptic vesicles [123,124,125] (see Table 3 for a summary of these genes).

The impairment of these molecular mechanisms can lead to dyshomeostasis of neuronal cells and ultimately cause neuron death.

Among the different genes linked to PD, mutations or gene duplication and triplication in the *SNCA* gene, which encodes the protein α-syn, are of particular interest. *SNCA* mutations typically lead to an increased α-syn propensity to aggregate or to form oligomers. Moreover, mutations can alter α-syn function at the presynaptic terminal by impairing its ability to bind lipid membranes and other interactors [126], which are crucial for its role in the regulation of synaptic vesicle recycling. Finally, this protein, likely in its aggregated forms, is involved in the spreading of the pathology across the body and different brain regions according to the Braak hypothesis [127], which would explain disease progression. Despite being still under debate, it is clear that monomeric and aggregated α-syn can be transferred between cells through extracellular vesicles and between organs, i.e., from the gut to the brain, thus contributing to inflammatory processes and neuronal damage as determined by different toxicity mechanisms. Besides causative mutations in specific genes, a large set of other mutations have been identified as risk factors for PD. These mutations are located on genes mainly involved in autophagic–lysosomal function and mitochondrial activity, but also in many other intracellular processes [128].

According to existing information, several animal models have been developed (reviewed in [129]) to study the molecular mechanisms associated with this disease and to test novel therapeutic strategies. In particular, most of the studies have relied on the use of either rat or mouse models, which carry mutations in the genes mostly relevant to genetic PD forms, or neurotoxin-based models, which are obtained by administering to the animals toxins that have been linked to PD in environmental studies.

Interestingly, most of the mutant mouse models do not recapitulate very well the disease phenotype, especially in terms of symptoms, neurodegeneration of specific brain areas, and the formation of α-syn inclusions, making them not always suitable for preclinical studies. On the other hand, neurotoxin-based models present the typical issues of acute disease models, with limited possibilities for the study of the progression of the disease. Experimental approaches based on the administration of different toxins to mice, i.e., rotenone or the neurotoxin 1-methyl-4-phenyl-1,2,3,6-tetrahydropyridine (MPTP), have been extensively used to study the role of sex hormones in PD, as discussed below, and their key features are summarized in Table 4.

### 2.2. Sex-Related Differences in Microglia and Their Implication on Neuroinflammation in PD

Neuroinflammatory processes are considered critical in the onset and development of neurodegenerative diseases. The molecular mechanisms of neuroinflammation have largely been characterized in recent years in relation to PD [118,130]. Their role in PD etiology and progression has been strongly validated based on post-mortem PD brain analyses and data from model organisms for this disease [131,132]. A description of these neuroinflammatory mechanisms (recently reviewed in [133,134]) is beyond the aim of this review; thus, we focus this section on sex differences in microglia, and the outcomes in the development of neuroinflammation in PD are described in the next section.

The innate and adaptive immune responses display sex differences, which development is regulated by genes, sex hormones, age, and reproductive state [135]. These differences in the immune responses could also explain, at least in part, the different susceptibility to neuroinflammation and sex prevalence in neurodegenerative diseases.

Microglia are considered the macrophages of the CNS: they can release reparative factors and adopt both pro-inflammatory and immunosuppressive phenotypes. Moreover, these cells regulate neural proliferation in early development by secreting pro-proliferative cytokines and further pruning superfluous cells by inducing apoptosis and targeted phagocytosis [78]. During adult neurogenesis, microglia control the plastic remodeling of neuronal circuits [136].

As reviewed by Chowen and Garcia-Segura in 2021, macro- and microglia show marked sexual dimorphism in their physiology and in their ability to react to brain pathology. For this reason, glial cells are recognized for being able to contribute in different ways to brain homeostasis and to the innate immune response which protects the brain against insults [137].

Microglia show different immunoreactive properties between the two sexes, which are initially established during sexual differentiation. The perinatal surge in testosterone and the hormonal milieu shape the morphology of different brain areas, including the microglial phenotype, which is further maintained during adulthood [78,138]. Moreover, the X chromosome contains a large number of genes related to the immune system, thus strongly contributing to the sex-related differences in immune response and inflammation (reviewed in [139]).

Male microglia have a higher density and higher phagocytic capacity, and they have been associated with more neuroinflammatory action, while female microglia are more supportive of neuronal functions, are neuroprotective, promote repair, and inhibit inflammatory response (reviewed in [140]). Interestingly, these abilities are also retained in the adult brain, as demonstrated by Villa and colleagues in 2018, who observed sex-related differences in the gene expression, in the morphology, and in the competence of female microglia to reduce ischemic damage even when transplanted in the male brain [138]. Adult microglial cells show sex-specific transcriptomes and are differentially subjected to the action of sex steroids depending on the hormone receptors they express (reviewed in [141]).

### 2.3. Sex Bias in Parkinson’s Disease and the Role of Sex Hormones

As in other neurodegenerative diseases, PD primarily affects men, with a ratio of 1,5:1, and post-menopausal women with a similar incidence. Female patients experience later emergence of motor symptoms, with a more tremorigenic phenotype and a slower disease progression compared to men, suggesting that male PD and female PD patients may present two disorders with very different features [115]. This evidence suggests that either sex hormones or genetic sex, or both, can contribute to the predisposition to and the development and progression of the disease by impacting those cellular pathways that are known to play a role in PD etiology.

#### 2.3.1. Estrogens

The sex difference in PD incidence points toward the possible protective effects of female steroid hormones, particularly estrogens. This possibility is also suggested by the reduced PD risk in women with longer exposure to endogenous estrogens due to early menarche and/or late menopause [142,143,144]. On the contrary, surgical or premature menopause, especially when occurring at an early age, is associated with an increased risk of PD [145,146]. However, besides these correlations, other studies have found no evidence of a positive effect of endogenous estrogens on the risk of PD [147,148], suggesting that the matter is likely more complicated.

Apart from epidemiological studies on PD incidence and its correlation with sex and female reproductive status, the neuroprotective and anti-inflammatory roles of estrogens have also been explored using model organisms of the disease.

Male rodents treated with the neurotoxin MPTP [149] show a higher depletion of striatal DA neurons and, consequently, of dopamine in the SN compared to females, thus mimicking the sex differences observed in human PD [150,151]. Similar results have recently been obtained using a rotenone animal model of PD [152].

The neuroprotective action of estrogens can also be due to the activation of anti-inflammatory pathways. In fact, estrogens have been found to modulate the response of microglia and astrocytes against oxidative and inflammatory injury in an MPTP rodent model, resulting in DA neuron protection [153]. Moreover, Tripanichkul and co-workers demonstrated that estrogen treatment prevents dopamine reduction, neuronal loss, and glial activation in the striatum of MPTP-treated animals and determines a decrease in the secretion of pro-inflammatory modulators [154]. A similar outcome was obtained with estradiol benzoate treatment in a MPP+ rat model of PD [155]. The authors also detected an upregulation of the expression of paraoxonase-2 (PON2), a mitochondrial protein working as an antioxidant. Interestingly, this protein is transcriptionally regulated by estrogens and presents higher expression levels in the brain of female mice, thus making female brain cells less sensitive to oxidative stress [156].

In a previous study, the anti-inflammatory action of estrogens was shown to also occur by reducing microglial reactivity in rodents treated with 6-hydroxydopamine (6-OHDA) [157]. In the same model, an induction of the autophagic process was found to be activated by estrogens to prevent degeneration of DA neurons [158].

Finally, by binding to the ERβ isoform, estrogens are known to decrease microglial activation [159]. Therefore, it is unsurprising that ovariectomy in mice leads to an increase in neuroinflammation [160].

The neuroprotective role of estrogens was confirmed using knockout mice for the *Cyp19a1* gene, which encodes the aromatase enzyme. These mice, that are unable to synthesize these hormones, were found to show dopaminergic neuron impairment and enhanced vulnerability to MPTP-induced nigrostriatal damage, when compared with both treated WT and even their ovariectomized counterparts. The latter result suggests that estrogens produced locally by the brain may fulfill important neuroprotective functions [161].

Estradiol also exerts its protective role by upregulating *BDNF* expression in the SN, as demonstrated in a 6-OHDA male rat model of PD [162].

Evidence shows that circulating estradiol upregulates the expression of TH, an enzyme responsible for dopamine synthesis [163]. Estradiol treatment, indeed, increases the fiber density of DA neurons in pharmacologically induced PD mouse models. This neuroprotective action seems to be specifically mediated by ERα [164], as confirmed by the higher vulnerability to MPTP in mice in which ERα has been silenced, compared to WT and ERβ KO mice [165,166]. This protective effect of estrogen through ERα in DA neurons requires the activation of membrane-bound G protein-coupled estrogen receptor (GPER) [167].

mERα can also exert a neuroprotective role by activating rapid non-genomic signaling cascades aimed at counteracting oxidative imbalance and mitochondrial dysfunction, thus promoting cell survival [168].

Finally, estradiol, besides being involved in neuroprotection as previously described, is thought to contribute to the maintaining of lipid rafts present in cell membranes, which provide a proper environment for optimal protein stability and molecular interactions on the cell surface. Aging and menopause-associated reduction in estrogen levels could lead to the loss of essential lipid components of membranes, finally causing neuronal dysfunction [169].

#### 2.3.2. Progesterone

Progesterone has been found to exert a protective effect on experimental models of PD. This hormone contrasts the DA neuron depletion induced by MPTP in male mice [170] and exerts neuroprotective effects on the striatal neurotransmission systems in a male rat PD model [171]. Interestingly, the myenteric plexus of the enteric nervous system in MPTP-treated mice benefits from progesterone, thus suggesting a role for this hormone in gut myenteric plexus defense and in the prevention of gastrointestinal alterations, which are one of the main non-motor features of PD [172].

Finally, the treatment with the progesterone metabolite, allopregnanolone, has been found to improve motor coordination and increase the expression of TH protein and TH cell number in a MPTP mice PD model [173].

#### 2.3.3. Androgens

The role of androgens in neuroinflammation and PD is less clear. PD patients have been reported to present reduced testosterone levels [174], thus suggesting a possible protective role of this steroid. Moreover, testosterone replacement therapy has been found to ameliorate motor symptoms of PD patients [175].

However, this hormone has recently been found to increase neuroinflammation in N27 dopaminergic cells under oxidative stress. This is shown to occur through a putative membrane-associated androgen receptor via the activation of inflammatory pathways (nuclear translocation of NF-κB and activation of COX2 signaling), which induces the apoptosis of DA neurons [176] as well as exerts a suppressive role in the midbrain dopaminergic pathways [177].

In good agreement with these findings, testosterone and DHT are unable to counteract MPTP-induced dopaminergic toxicity [178,179].

In contrast with the results described above, the positive effects of chronic treatment with testosterone propionate, such as improved motor problems and reduced dopamine depletion, have recently been reported in a reserpine-induced progressive rat model of PD [180].

Unfortunately, we were unable to find studies reporting the outcome of sex hormone treatment in PD genetic animal models, which would be of great interest to understand the possible role of hormone-based therapies in different disease models. This could be ascribed to the limited ability of genetic PD animal models to decently recapitulate the disease phenotype, so this piece of information remains unavailable.

### 2.4. Effects of Estrogen-Based Pharmacological Treatments on Parkinson’s Disease

Exogenous sex steroid hormone therapies (oral contraceptives, supplemented estrogens and progestogens, and synthetic anabolic steroids) are largely used to treat different conditions, such as testosterone deficiency (hypogonadism) [181], contraception or postmenopausal symptoms [182].

Due to the pleiotropic functions that sex steroid hormones exert on the brain during both development and adult life, the therapeutic use of these steroids can affect the brain in different ways. Therefore, it is not surprising that hormone replacement therapies (HRT) can impact cellular processes and pathologies involving this organ.

Regarding postmenopausal hormone therapies, their use has been reported to lead to a reduced risk of developing PD or a diminished disease severity together with an improvement in DA activity and dopamine transporter density [145,183]. Still, the results reported in different studies regarding PD incidence and the possible positive effects of HRT on this disease remain controversial, likely because of the different factors (dosage, duration, and composition of the replacement therapies used) that have been considered in each study [184,185,186].

In different neurotoxin animal models of PD, protective effects have been obtained when treating the animals with estrogens, as well as ER modulators (SERMs) or ER agonists (reviewed in [187]), thus suggesting that a potential repositioning of steroids, particularly estrogens, may improve the life quality of PD patients [186]. Unfortunately, estrogen replacement therapies can also be associated with detrimental peripheral side effects, such as uterine and breast stimulation, and consequent increased risk of cancer in these organs, stroke, coronary heart disease, and vascular problems [188]. These complications can be overcome by using estrogens designed to act only in the brain, where they can positively affect neurological diseases and menopausal symptoms, such as hot flushes, depression, and cognitive impairment, while sparing the adverse peripheral side effects.

Recently, Prokai and co-workers [189] synthesized and characterized a small-molecule bio-precursor prodrug, the “10b,17b-dihydroxyestra-1,4-dien-3-one”, named DHED. After its systemic administration, DHED is rapidly converted to 17β-estradiol, preferentially in the rat brain, where it stimulates the same gene expression and neuroprotective effects obtained with 17β-estradiol treatment [190].

In vitro metabolic studies have demonstrated that DHED conversion to active estrogens does not occur in estrogen-sensitive peripheral tissues. Interestingly, this molecule reduces symptoms associated with the loss of brain estrogens and results in neuroprotective effects in rats [189,190]. In addition, this molecule cannot bind to ERs and, due to its physicochemical characteristics (increased water solubility and reduced binding to plasma proteins compared to 17β-estradiol), it crosses the blood–brain barrier more easily, thus showing a better uptake in the brain [190].

This prodrug has been tested in a double-transgenic mouse model of Alzheimer’s disease in a side-by-side comparison with 17β-estradiol [191,192]. The authors reported that the DHED therapy achieved the same positive neuro-biochemical effects and behavioral improvement obtained with the 17β-estradiol treatment.

The potential of this prodrug has also been analyzed in PD models. In a symptomatic α-syn mouse model carrying the E46K human PD mutation in the *SNCA* gene, the brain increase in estrogens after DHED administration reduced PD-like neuropathy and improved behavioral effects in both female and male mice [193]. In a MPTP-induced PD mouse model, DHED treatments reduced the behavioral impairment and degeneration of DA neurons in the striatum and in the SN. Moreover, the authors measured a decrease in α-syn monomer accumulation and aggregation and a reduction in the levels of oxidative and inflammatory markers [194].

Although requiring more investigations, the lack of peripheral side effects and its brain-specific estrogenic activity suggest that this prodrug could represent an attractive potential therapeutic approach for neuroprotection and treatment of neurodegenerative disorders, including PD.

### 2.5. Contribution of Genes Located on the Sex Chromosomes to Parkinson’s Disease Etiology

Other possible sex-related contributors to the etiology of PD, which may explain the prevalence of male PD patients, could be the genes expressed on the X and Y chromosomes.

Besides the obvious absence of Y-chromosome genes in XX individuals, it is well-established that the expression levels of X-linked genes can vary between males and females due to epigenetic marks and because some of these genes can be expressed from both gene copies in XX cells, leading to a higher genetic load compared to the XY genotype [195]. In fact, the XCI can occur, but it may not always be able to level the differences. Interestingly, the X chromosome contains a six-fold greater number of genes involved in neurodevelopmental and neurophysiological processes than autosomes [195]. Moreover, in XY individuals, the maternal inheritance of the X chromosome leads to a single possible imprinting of X-linked genes, which cannot be balanced as it happens for females. This evidence further suggests that the differential expression of some of the X- or Y-linked genes can occur. Finally, mutations in X-linked genes have been associated with different disorders that are often characterized by intellectual disability, such as the Rett syndrome, the fragile X syndrome, and the Börjeson–Forssman–Lehmann syndrome [195].

In the PD framework, X-linked forms of parkinsonism have been reported and are associated with mutations in different genes (see Table 3). One example is the *RAB39B* gene, which encodes the small GTPase RAB39B and is located on the X chromosome. A variety of *RAB39B* mutations have been found, which mainly cause protein truncation, destabilization, or mislocalization, and are linked to early onset PD with intellectual disability [196,197]. This is believed to be associated with defects in the autophagic pathway, which were found to impact on synapse formation and function in *Rab39B* knockout mice [198].

Along the same line, it has been shown that about 39% of the male carriers of the expanded CGG repeats of the fragile X mental retardation gene (*FMR1*) present fragile X-associated tremor/ataxia syndrome (FXTAS) [199]. FXTAS patients often present PD or parkinsonism, suggesting a possible link between the two diseases [200]. The occurrence of PD pathological hallmarks, such as the occurrence of protein inclusions in the brain, in FXTAS patients suggests that *FMR1* can actually be considered a PD-associated gene [201].

Mutations in the *TAF1* gene, which is located on the X chromosome, have been associated with X-linked dystonia-parkinsonism (XDP) [202]. TAF1 protein is involved in the regulation of the initiation of transcription by RNA polymerase II, and it is still unclear how it participates in the development of XDP. Nevertheless, XDP is primarily observed in males [203], while no association with *TAF1* variants and female PD patients has been identified, which may be caused by the pattern of XCI previously mentioned [202].

Another interesting example is represented by a rare polymorphism in the X-linked gene, *GLUD2*, which encodes a mitochondrial glutamate dehydrogenase specifically expressed in the brain. Gain-of-function mutations in this gene result in an earlier age of onset in PD, which does not occur in heterozygous female patients with PD, likely because estrogens are known to suppress its enzymatic activity thus compensating for the aberrant enzymatic activity caused by the mutations [204]. This estrogen effect on GLUD2 activity would further suggest that the interplay between genetic sex and sex hormones also needs to be taken into account when investigating molecular mechanisms associated with PD in males and females.

In all existing cases, limited information is available to understand how X-linked mutations in parkinsonism-associated genes may differentially contribute to the molecular mechanisms underlying the etiology of the disease. In an attempt to further verify the specific contribution of mutations on genes located on the X chromosome, X-chromosome-wide association studies (XWAS) would be recommended. Nevertheless, even if the X chromosome accounts for about 5% of the human genome and would provide interesting information on the matter discussed in this part of the review, it is usually excluded from most GWAS studies because of technical difficulties, thus hampering the access to this information [205]. The first report providing a XWAS on PD patients was obtained through a meta-analysis that included all available PD cohorts with data on the X chromosome, with the goal of identifying possible sex-specific risk factors that were not observed before [205]. In this study, two novel genome-wide significant loci were found, rs7066890 and rs28602900, with the latter linking the *RPL10* gene to PD, whereas it was formerly only associated with complex forms of intellectual disability with epilepsy [206].

To further examine the possible contribution of genes present on the sex chromosomes to PD, we considered a subset of genes located on the X or Y chromosome known to be involved independently in the brain sexual differentiation regardless the levels of sexual hormones [195]. Among these genes, some are associated with alterations in the functioning of the dopaminergic system and/or with altered proteostasis of α-syn, which are two of the key hallmarks of PD. This suggests that they may play a role in the predisposition to PD or contribute to disease progression or severity when their expression is altered.

We identified some X-linked and Y-linked genes that deserve to be examined thoroughly, but others may need attention in the future. For example, one interesting candidate is the X-linked *MECP2* gene, which encodes Methyl-CpG-binding protein 2 (MeCP2) and is involved in the regulation of gene expression through various mechanisms. Interestingly, *MECP2* is typically associated with a neurodevelopmental disorder called the Rett syndrome, which affects almost exclusively females and can present motor symptoms very similar to those observed in PD patients. In this framework, it was shown that the loss of the *Mecp2* gene in a Rett syndrome mouse model led to the impairment of the nigrostriatal pathways and motor deficits, which include reduced dopamine release after striatal stimulation and defects in the D2 dopamine receptor-dependent electrophysiological activity of DA neurons [207]. A recent transcriptome analysis performed on *Mecp2* knockout mice showed that they present alterations not only in development-related pathways but also in additional pathways that are more generally associated with other neurological diseases, such as defects in neuronal transmission and immune reactivity [208], both of which are important in PD.

When considering other X-linked genes within this subgroup, two additional genes reveal interesting hints for their possible link with PD development, i.e., *USP9X* encoding the protein ubiquitin-specific peptidase 9 X-linked and *OGT* encoding OGlcNAc transferase, both of which have been associated with intellectual disabilities [209,210].

In the PD framework, these proteins are particularly interesting because both ubiquitination and O-GlcNAcylation, together with phosphorylation and oxidation, are key posttranslational modifications of α-syn that allow the maintenance of its balance and homeostasis in cells [126,211]. USP9X was shown to deubiquitinate α-syn, but USP9X levels were significantly lower in the cytosolic fractions of the SN of PD patients compared to controls [212]. According to the authors, this may contribute to the accumulation of monoubiquitinated α-syn in Lewy bodies, which also occur in vitro when *USP9X* is downregulated.

In females, *USP9X* expression could be higher compared to males because *USP9X* is a gene known to escape X inactivation [213], providing a possible protective effect that reduces α-syn aggregation and, thus, the PD incidence in the female population.

Something similar may occur for the *OGT* gene: a higher expression in females may increase O-GlcNAcylation levels of α-syn. Since α-syn O-GlcNAcylation is shown to have site-specific inhibitory effects on α-syn aggregation and toxicity in vitro and in cell models [214] and in adenovirus α-syn mouse models for PD [215], *OGT* expression levels may be protective in XX individuals compared to XY individuals. It is worth noting that O-GlcNAcylation is generally crucial in neuronal development and signaling. Specifically, increased O-GlcNAcylation improves synaptic function in DA neurons [215], likely by reducing α-syn toxicity. However, other effects supporting DA neuronal function cannot be excluded.

Of note, male-specific genetics could also contribute to males’ higher susceptibility to PD. For example, the *SRY* gene is expressed in male nigrostriatal dopaminergic neurons, which are more numerous in males than females, and they are known to be involved in dopamine synthesis and metabolism [216]. It has been reported that misregulation of this protein could contribute to PD development. Czech and colleagues in 2012 and 2014 demonstrated an increase in *SRY* expression in response to toxin-induced insults in male DA neurons and in a male neuroblastoma cell line [106,217], whereas Lee et al. (2019) correlated the reduction in SRY expression in toxin-induced rat models for PD (6-ODHA and rotenone models) with a decreased loss of DA neurons and with ameliorated motor defects. This occurred through an improvement in mitochondrial defects and inflammation induced by the toxin administration and, thus, suggested that *SRY* inhibition might be a male-specific therapeutic strategy in PD. According to the authors, these seemingly contrasting results can be explained by the in vitro system that lacks the cellular network between DA neurons and microglia and by the different time course of *SRY* upregulation [218].

Finally, the Y-linked *NLGN4Y* gene is also reported to be a key for sexual brain development and relevant for the discussion. This gene encodes a membrane protein that is part of the neuroligin family, which includes cell adhesion proteins localized at the postsynaptic side of synapses and are crucial for synapse formation [219]. As previously discussed, synaptic defects are one of the molecular mechanisms associated with PD etiology [220].

Interestingly, a similar X-linked *NLGN4X* gene, which encodes a protein with 97% of sequence identity with the neuroligin encoded by *NLGN4Y*, has been suggested to have the same function. *NLGN4X* variants, which have been associated with autism [221], display defects in protein trafficking and in induced synaptogenesis similarly to NLGN4Y protein. This may suggest that in male PD patients, the expression of a less functional form of neuroligin may predispose these patients to the formation of less stable or less functional synapses, contributing to PD etiology or to some of the manifested symptoms.

### 2.6. Sex Differences in Immortalized and Primary Cells Relevant to Parkinson’s Disease Research

In light of what we have reported in this review, it appears clear the importance of taking into account the biological sex of the models used in scientific research, both when using animal models but also when considering cell lines. As shown in different reports, the sex of the animals used is often omitted, and a strong bias is observed toward the use of males. The genetic sex of cells is stated only in a minority of the published journal articles [222,223]. This situation has started changing in recent years, with an increase in the percentage of papers that comment on the sex of the model used. Kim and collaborators in 2018 reported that, among relevant manuscripts published in *AJP-Cell Physiology*, 50% indicated the sex of the cells used: a promising trend if compared with what was observed by Shah and collaborators in 2013, when the same journal could not boast more than 25% of papers assessing the sex of the cells used. However, between 2021 and 2022, this percentage dropped again (as reviewed in [224]). Investigators analyzing other neuroscience journals found less than satisfying rates of sex reporting (for a more detailed description of this phenomenon refer to [222,223,224,225,226]), and most of the existing studies failed to consider sex as a relevant biological variable even when both male and female models were used. This approach leads to an underestimation of possible sex differences due to different responses to drug treatments or genetic manipulations [227,228]. Interestingly, studies dealing with primary human cells were less precise on their model sex compared with those on non-human animal cells, and among the latter studies, cells isolated by researchers were better described than the purchased ones [222].

Cells do have biological sex that is intrinsically determined by the genetic complement of genes encoded by the X and Y sexual chromosomes and by the regulation of epigenetic mechanisms, all independent from the action of hormones, as evidenced, for example, by the morphological and functional differences observed in DA neurons derived and cultured from rats at embryonic day 14 (E14), while gonadal release of testosterone only starts from E15 onward [229].

Sex-related differences have been reported in cells of almost all tissues, including the brain [78,137,223]. Other than gene expression, male- and female-derived neuronal cells differ in morphology; growth rate and maturation; neurotransmitter metabolism; and response to insults, such as starvation, oxidative stress, and inflammation [137,223,230].

This is again of pivotal importance when dealing with the study of diseases characterized by a sex bias, as it happens in many neurodegenerative diseases, including PD.

The sex of cells and cell lines could be determined based on an analysis of the expression profile of the amelogenin gene, which is encoded by both X and Y chromosomes (*AMELX* and *AMELY*, respectively) and can be easily distinguished by using PCR because they present different lengths [231]. However, the sex of cells may change over time or after several passages in culture, even in established cell lines [224]. Cells derived from female donors may present chromosome Y fragments, while male-derived cells can completely lose their Y chromosome; thus, they can no longer be univocally assessed for their sex [223,232]. Moreover, different cell stocks, laboratory protocols, and culture conditions may contribute to the genetic variability and diversification among cell lines [224].

Beyond the intrinsic, chromosome-dependent sex, it is important to consider the hormonal environment where cells are grown. Cell culture media can indeed influence the hormonal milieu with estrogen-like compounds, which may differentially influence male and female cells. Serum contains all biomolecules and growth factors necessary for cell growth, cell metabolism, and cell attachment and proliferation, but it is usually rich also in hormones, including sex steroids. Phenol red, which is a common pH indicator present in most commercially available culture media, has been shown to bind and activate ERs, leading to estrogen-like effects in different cell types, including neurons, and even plastic materials could have weak estrogenic outcomes from leached phenolic compounds [226,232].

Therefore, the sex of cells is a matter that deserves attention and must be considered to provide as much complete information as possible, thus taking a first step toward considering and including sex as a biological variable. This is especially important from the perspective of tailored medicine that can address the differences observed between male and female patients.

As PD is one of the most frequent neurodegenerative diseases that affects the elderly population worldwide, with substantial human and social costs, it is necessary to address the molecular causes underlying its occurrence and the mechanisms of its progression to develop new strategies to counteract the neuronal degeneration and introduce new therapies. To do so, researchers are prompted to develop the most suitable models that better recapitulate the many features characterizing this complex disorder. As previously reported, PD shows a strong sex bias, affecting primarily males. Sex contribution, therefore, should be considered in the model used and monitored as a fundamental variable of disease outcome and progression.

The main cellular lines used to study PD are the well-established human neuroblastoma cell line SH-SY5Y and the lesser-characterized BE(2)-M17 [233].

The first one is a subline of the SK-N-SH cell line derived from the bone marrow biopsy of a metastatic neuroblastoma in a 4-year-old female. It was cultured in 1970 and underwent three rounds of clonal selection [234]. BE(2)-M17 cells, instead, were derived from a SK-N-BE(2) neuroblastoma cell line established in 1972 from the bone marrow tumor of a 2-year-old male patient.

They can both differentiate into neuron-like cells upon differentiation protocols with the administration of retinoic acid (RA). However, it has been reported that while RA is suited for obtaining valuable BE(2)-M17 neuronal morphology and functionality, in the SH-SY5Y cell line the most evident effects are induced after treatment with staurosporine [233,235].

It has been observed that these two cell lines show different features at the catecholaminergic level, with the BE(2)-M17 cell line having a more prominent dopaminergic phenotype, compared with the SH-SY5Y cell line, which expresses a more noradrenergic phenotype [233,235].

BE(2)-M17 cells show a more pronounced expression of TH and other markers associated with dopamine metabolism, such as DAT, a specific dopamine transporter, and VMAT2, a monoamine transporter that loads dopamine, serotonin, and other neurotransmitters into synaptic vesicles. Upon differentiation, all of these dopaminergic markers increase their expression. SH-SY5Y cells instead mildly express TH only when differentiated with staurosporine and present an increase in dopamine-ß-hydroxylase [236], which converts dopamine to noradrenaline and is specific of noradrenergic neurons [235,236]. Moreover, differentiation induces an increase in VMAT2 and SERT, a specific serotonin transporter, as it would be expected by serotonergic neurons [233]. Therefore, as it has been reported in different papers [233,235,236], the use of the BE(2)-M17 cell line could be more suitable to characterize the molecular mechanisms of PD that directly involve dopamine metabolism.

The genetic sex of these cells could provide other valuable information on the disease. Czech and collaborators verified that male BE(2)-M17 cells treated with toxin 6-OHDA, a product of dopamine metabolism that is involved in the generation of ROS and consequent DNA damage, present an increase in the expression of *SRY* mRNA and SRY protein transcription. This upregulation occurs rapidly and has a protective role in reducing reactive oxidative species and apoptosis [217]. However, SRY expression is reduced upon prolonged treatment and chronic neuronal injury, and its defensive role is, thus, ablated. Intriguingly, knockdown of endogenous SRY expression in BE(2)-M17 cells leads to increased cell damage and oxidative stress, whereas ectopic over-expression of *SRY* in treated feminine SH-SY5Y cells appears to be protective [217]. Human immortalized cell lines are valuable tools to dissect the molecular mechanisms that characterize PD, but they bring disadvantages, such as their oncogenic origin, which prevents them from fully recapitulating some key functional neuronal properties [237].

Other in vitro models are widely used, such as primary rodent cultures derived from different brain regions. Cultured DA neurons maintain morphological and physiological properties and produce the same receptors and transmitters as in vivo [237]. Interestingly, primary cultures display sex differences regarding growth rate, proliferation, and gene expression [227,238]; therefore, the sex of the animals used should be considered. Among the possible drawbacks of this model, as reviewed by Ferrari et al. (2020), there is the heterogeneity of the obtained cell population, with the simultaneous presence of different cellular types from the glia, and the fact that these cells can only divide a finite number of times. Moreover, they lack human genetic background [237].

The generation of human-induced pluripotent stem cells (iPSCs), after the discovery of this powerful technology by Takanshi and Yamanaka in 2006 [239], and the recent possibility of obtaining midbrain organoids overcome some of these issues.

iPSCs show a mature morphological and physiological phenotype, and they can also be derived from patients with known biological sex bearing different forms of familial or idiopathic PD. iPSC-derived DA neurons can be obtained [240], presenting the limitations of 2D cultures. More recently, 3D midbrain organoids have also been developed. These human-derived systems can model existing neuronal circuits and allow further analyses to better understand the interactions between neurons and glia in a mature physiological environment, thus providing a precious tool to study such a multifaceted and complex disease [237].

## 3. Conclusions

Altogether, the studies discussed in this review suggest that not only sex hormones but also genetic sex and epigenetic factors are key elements in brain development and physiology. The resulting sexual dimorphism seems to be connected to the predisposition toward and etiology of different neurodegenerative diseases.

Even if aging is a major known risk factor for neurodegenerative disorders, several pieces of evidence suggest that the contributions to their development may come from processes that occur earlier in life [241]. Thus, it is tempting to speculate that sex-related differences in brain development described in the first part of this review may already play a role in the etiology of all neurodegenerative diseases that occur not earlier than midlife.

In this paper, we have focused our attention on PD, one of the most frequent neurodegenerative pathologies that shows strong sex-related prevalence.

X-linked forms of parkinsonism are reported, and genes located on the sex chromosomes and involved in brain development may also have a key role in certain molecular mechanisms relevant to PD progression. At the same time, sex hormone levels seem to be connected to the incidence of PD, and estrogens have even been proposed as a possible alternative therapeutic strategy.

Based on the literature reviewed in this work, the study on PD etiology, progression, severity, and symptoms requires two major improvements in the future: (i) pre-clinical studies should clearly take into account the sex of the model in use, not only at the level of animal models, but also when using immortalized cell lines and primary cell cultures; (ii) therapeutic strategies specifically addressing male and female patients should be proposed, and clinical trials for biomarker evaluation and drug testing should be designed accordingly.

The topic we addressed in this review is wide and multifaceted, spanning from the role of biological sex in brain development to its contribution to the outcomes of neurodegenerative diseases. The interplay between hormones and genetic background is complex. Moreover, not only PD but also many other neurodegenerative disorders display sex prevalence. Thus, we could not cover all the aspects that may be relevant in this large framework. However, with this work, we aim to increase awareness on the importance of considering sex as a fundamental variable in the study of the molecular mechanisms defining brain physiology, pathophysiology, and neurodegenerative diseases.

## Figures and Tables

**Table 3:** Genes mainly involved in Parkinson’s disease etiology.

Gene	Gene Product	Function	PD Inheritance
**Well established**			
*SNCA*	Alpha-synuclein	Synaptic vesicle exocytosis; DA neurotransmission; chaperone activity	Dominant
*LRRK2*	Leucine-rich repeat kinase 2	Neuronal plasticity; autophagy; vesicle trafficking; neuroinflammation	Dominant
*VPS35*	Vacuolar protein sorting 35	Membrane protein recycling	Dominant
*PRKN*	Parkin	Mitochondrial quality control	Recessive
*PINK1*	PTEN-induced kinase 1	Mitochondrial quality control	Recessive
*DJ-1*	DJ-1	Protection against oxidative stress; mitochondrial function	Recessive
**Atypical complex PD**			
*ATP13A2*	Cation-transporting ATPase 13A2	Lysosomal cation and polyamine transporter	Recessive
*DNAJC6*	DNAJ subfamily C member 6	Endocytosis of clathrin-coated vesicles	Recessive
*FBXO7*	F-box protein 7	Ubiquitination; proteasome degradation	Recessive
*SYNJ1*	Synaptojanin-1	Synaptic vesicle endocytosis; actin filament rearrangements	Recessive
*PLA2G6*	Phospholipase A2, group 6	Membrane homeostasis; mitochondrial integrity; signal transduction	Recessive
**Risk factors**			
*GBA1*	Glucosylceramidase beta	Lysosomal function; lipid metabolism	Risk factor
*MAPT*	Microtubule-associated protein Tau	Axonal stability; axonal transport	Risk factor
**X-linked genes associated with parkinsonism**			
*RAB39B*	Ras-related protein Rab-39B	Vesicular trafficking	X-linked parkinsonism
*FMR1*	Fragile X messenger ribonucleoprotein 1	Synaptic plasticity; negative role in translation	FXTAS with parkinsonism
*TAF1*	TATA-box-binding protein associated factor 1	Initiation of transcription	X-linked dystonia-parkinsonism
*GLUD2*	Glutamate dehydrogenase 2	Glutamate metabolism; neurotransmission	Polymorphism

**Table 4:** Neurotoxin-induced animal models for Parkinson’s disease.

PD Model	Effect	Phenotype
Acute MPTP model	Inhibition of complex I	Motor deficit; DA neuron death; No α-syn aggregates.
Subacute/chronic MPTP model	Inhibition of complex I	Progressive model; No motor deficit; No DA neuron death; α-syn aggregates.
6-OHDA	Inhibition of complex I and oxidative stress	Asymmetric motor deficit; DA neuron death; No α-syn aggregates.
Rotenone	Inhibition of complex I	Limited motor deficit; Moderate DA neuron death; α-syn aggregates.
Paraquat	Oxidative stress	Limited motor deficit; Limited DA neuron death; α-syn aggregates.

## Data Availability

Not applicable.
